# Histone Demethylase KDM4C Is Required for Ovarian Cancer Stem Cell Maintenance

**DOI:** 10.1155/2020/8860185

**Published:** 2020-08-29

**Authors:** Guo-Qing Chen, Ping Ye, Rong-Song Ling, Fa Zeng, Xiong-Shan Zhu, Lu Chen, Yan Huang, Ling Xu, Xiao-Ying Xie

**Affiliations:** ^1^Department of Obstetrics, Affiliated Shenzhen Maternity & Child Healthcare Hospital, Southern Medical University, Guangdong, China; ^2^Department of Obstetrics and Gynecology, First Affiliated Hospital of Gannan Medical University, Jiangxi, China; ^3^Institute for Advanced Study, Shenzhen University, Shenzhen, China

## Abstract

Ovarian cancer is a highly deadly disease, which is often diagnosed at a late stage with metastases. However, most ovarian cancers relapse after surgery combined with platinum-based chemotherapy. Cancer stem cells (CSCs) are stem-like cells that possess high tumorigenic capability and display higher resistant capability against current therapies. However, our knowledge of ovarian CSCs and their molecular mechanism remains sparse. In the current study, we found that KDM4C, a histone demethylase, was required for ovarian cancer stem cell (CSC) maintenance. Depletion of KDM4C significantly reduced the CSC population and sphere formation in vitro. Moreover, we found that KDM4C can regulate the expression of stem cell factor OCT-4 via binding to its promoter. These data indicate that KDM4C is relevant for ovarian CSC maintenance and underscore its importance as a potential therapeutic target.

## 1. Introduction

Ovarian cancer has the highest mortality rate of any gynecological malignancies around the world [1]. Ovarian cancer has an asymptomatic onset, and the majority of cases diagnosed are in the late stages with metastasis. Currently, surgery and chemotherapy are the main treatments for ovarian cancer, whereas a large majority of patients with advanced ovarian cancer relapse due to therapy resistance [2]. To overcome this dilemma in ovarian cancer, it is important to understand the molecular mechanism underlying therapy resistance of ovarian cancer [3]. The recently proposed hypothesis of cancer stem cells (CSCs), also known as tumor-initiating cells, may provide a more effective approach for the treatment of ovarian cancer. CSCs are stem-like cells that possess the tumorigenic capability to self-renew and differentiate into multiple cell types [4]. Moreover, CSCs display higher resistant capability against current therapies and are believed to be responsible for tumor metastasis [5, 6]. Hence, understanding of the molecular regulation mechanism of CSCs might provide new targets for treatment of ovarian cancer.

In recent years, histone modifications, such as histone methylation and acetylation, are emerging as critical mechanisms to regulate gene expression [7]. It plays an important role for coordination and organization of the chromatin structure during a variety of biological processes such as DNA replication, repair, and transcription [7]. Histone methylation status has also been involved in induced pluripotent stem (iPS) cell reprogramming by expression of OCT-4 and Sox2 [8]. In many tumors, changes of histone demethylase expression have been identified as a key characteristic during cancer initiation and progression, suggesting that these genes might be functionally important for cancer development [9]. Indeed, histone demethylases, such as KDM3A and KDM6A, have been shown to be essential for maintenance of CSCs in several types of cancers [10–13].

Deregulation of KDM4C, a H3K9me3 and H3K9me2 demethylase [14], has been identified in several solid tumors, such as esophageal squamous carcinoma, lung cancer, pancreas cancer, and breast cancer [15–18]. In this study, we used the sphere culture approach to enrich the CSC population of ovarian cancer and found that KDM4C is upregulated in the tumospheres. Furthermore, we found that depletion of KDM4C inhibits the migration, invasion, and CSC properties of ovarian cancer cells. Finally, our data revealed that KDM4C can regulate the expression of stem cell factor OCT-4 via binding to its promoter. In summary, we identify a crucial role of KDM4C in the maintenance of CSCs in ovarian cancer.

## 2. Results

### 2.1. KDM4C Is Upregulated in the CSC Population in Ovarian Cancer Cell Lines

**Figure 1:**
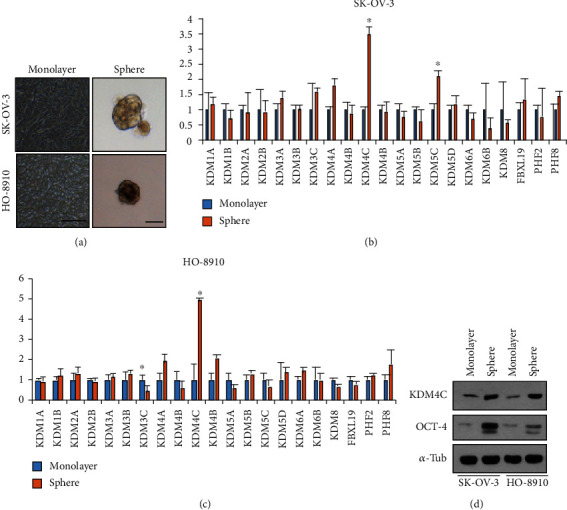
KDM4C is elevated in the tumorsphere of ovarian cancer cell lines. (a) Representative images of the monolayer and spheres of SK-OV-3 and HO-8910 cells. Scale bar, 100 *μ*m. (b) The mRNA expression of histone demethylases in the monolayer and spheres of SK-OV-3 cells was assessed by real-time RT-PCR. (c) The mRNA expression of histone demethylases in the monolayer and spheres of HO-8910 cells was assessed by real-time RT-PCR. (d) Western Blot analysis of the monolayer or spheres of SK-OV-3 and HO-8910 cells for KDM4C and OCT-4 expression. *α*-Tubulin is used as the control. ^∗^*P* < 0.05.

Cancer stem cells display greater ability to form spheres when placed in low attachment conditions in defined serum-free media (Figure 1(a)). To investigate the epigenetic regulation in ovarian cancer cells, we first performed a qRT-PCR assay to detect the transcription level of 21 histone demethylases in SK-OV-3 ovarian cancer cells grown under adherent or sphere-forming conditions (Figures 1(a) and 1(b)). Our results demonstrated that KDM4C and KDM5C were significantly increased in SK-OV-3 spheres, while the rest of the histone demethylases showed comparable mRNA expression levels (Figure 1(b)). We observed a similar elevation of KDM4C mRNA expression and a significant decrease of KDM3C expression when comparing the adherent and sphere culture of HO-8910 ovarian cancer cells (Figure 1(c)). Together, our data suggested that KDM4C might play a role in ovarian CSC maintenance. In addition, Western Blot results showed that the KDM4C protein level was increased in the SK-OV-3 and the HO-8910 sphere when compared with the monolayer culture (Figure 1(d)). Consistent with previous reports, stem cell factor OCT-4 was also enriched in these CSCs (Figure 1(d)) [19].

### 2.2. Downregulation of KDM4C Inhibits Cell Migration and Invasion of Ovarian Cancer Cells

**Figure 2:**
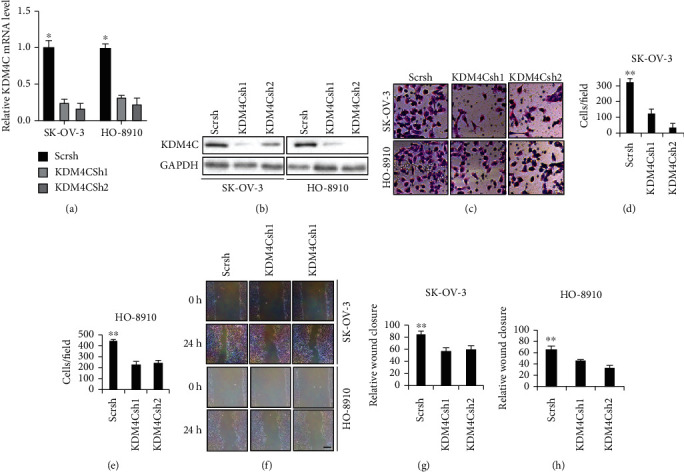
KDM4C is required for the invasion and migration ability of ovarian cancer cells. (a, b) The knockdown of KDM4C by shRNA. KDM4C expression was examined by qRT-PCR and Western Blot analysis. Scramble shell (Scrsh) was used as the control. (c, d) Cell invasive ability of SK-OV-3 and HO-8910 cells determined by transwell assays was decreased after stable knockdown of KDM4C. (e, f) Migratory potential of SK-OV-3 and HO-8910 cells determined by wound-healing assays was decreased after stable knockdown of KDM4C. Scale bar, 100 *μ*m. ^∗^*P* < 0.05, ^∗∗^*P* < 0.01.

Next, we sought to investigate the function of KDM4C in the ovarian CSCs by generation of two stably transfected KDM4C shRNA in SK-OV-3 and HO-8910 cells. Both cell lines transfected with shRNA showed dramatic inhibition of KDM4C mRNA and protein levels compared with the cells transfected with scramble control shRNA (Figures 2(a) and 2(b)). MTT assay results showed that inhibition of KDM4C did not affect the cell proliferation of SK-OV-3 and HO-8910 cells (Figure S1). Transwell assay results showed that KDM4C downregulation significantly reduced the invasiveness of SK-OV-3 and HO-8910 cells (Figures 2(c)–2(e)). In addition, depletion of KDM4C led to inhibition of the migration ability of SK-OV-3 and HO-8910 cells (Figures 2(f)–2(h)).

### 2.3. Downregulation of KDM4C Inhibits CSC Properties of Ovarian Cancer Cells

**Figure 3:**
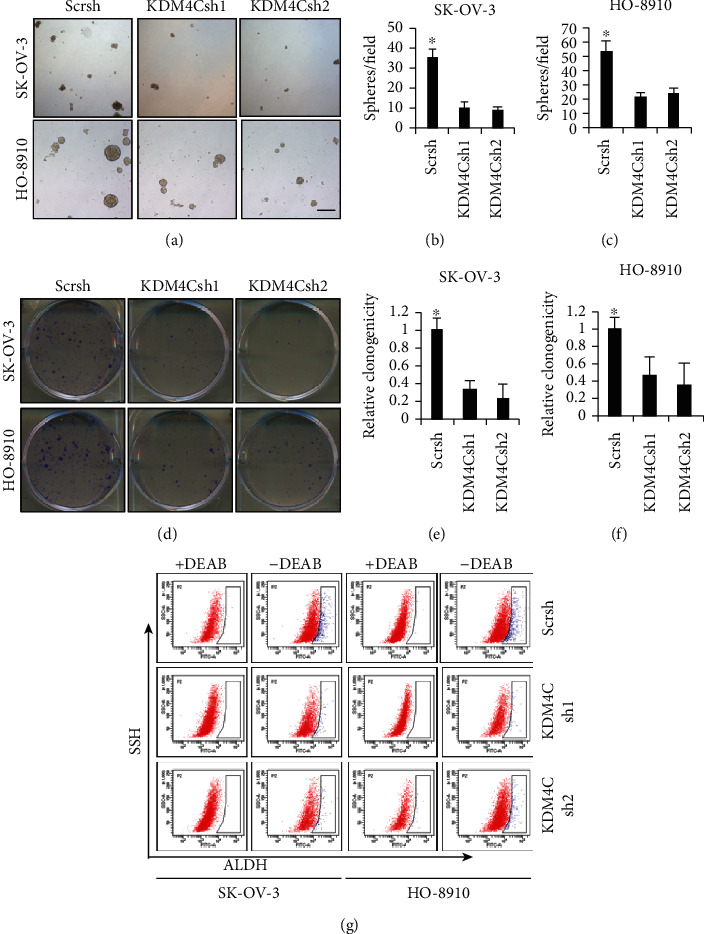
KDM4C is required for stem cell properties of ovarian cancer cells. (a–c) Sphere formation ability of SK-OV-3 and HO-8910 cells was inhibited after KDM4C depletion. Scale bar, 100 *μ*m. (d–f) Colony-forming ability was decreased after knockdown of KDM4C in SK-OV-3 and HO-8910 cells. (g) The percentage of the ALDH^high^ population in SK-OV-3 and HO-8910 cells was reduced after knockdown of KDM4C. ^∗^*P* < 0.05.

Sphere formation and colony formation assays were performed to examine the effect of KDM4C on CSC properties of SK-OV-3 and HO-8910 cells. Our results revealed that the sphere-forming capacity was markedly diminished after depletion of KDM4C in SK-OV-3 and HO-8910 cells (Figures 3(a)–3(c)). In addition, cell colonies were significantly reduced after KDM4C knockdown in SK-OV-3 and HO-8910 cells (Figures 3(d)–3(f)). Furthermore, we evaluated the aldehyde dehydrogenase (ALDH) activity, a known stem cell feature, of ovarian cancer cells after KDM4C knockdown using flow cytometry. The results showed that the percentage of the ALDL^high^ population was reduced in the KDM4C knockdown cells compared with the control (Figure 3(g)). Altogether, these results indicated that KDM4C is required for maintenance of CSC characteristics in ovarian cancer cells.

### 2.4. KDM4C Regulates OCT-4 Gene Expression via Binding to Its Promoter

**Figure 4:**
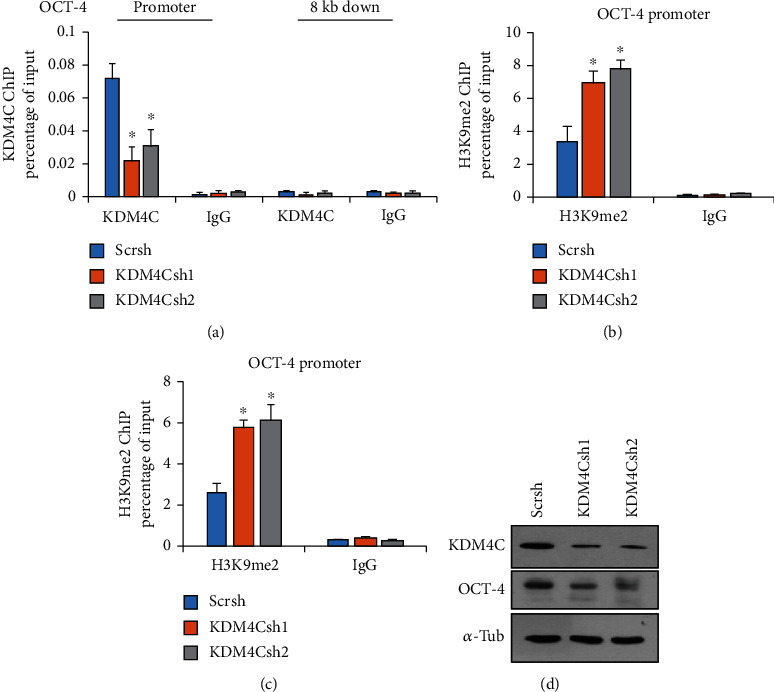
Increased KDM4C occupancy and H3K9 methylation levels at the OCT-4 gene promoter. (a) ChIP analysis of KDM4C occupancy at the OCT-4 promoter after KDM4C inhibition as quantified by real-time PCR. (b) ChIP analysis of H3K9 dimethylation levels at the OCT-4 promoter. (c) ChIP analysis of H3K9 trimethylation levels at the OCT-4 promoter as quantified by real-time PCR. (d) Western Blot analysis of SK-OV-3 spheres for KDM4C and OCT-4 expression. *α*-Tubulin is used as the control. ^∗^*P* < 0.05.

KDM4C-mediated H3K9me3 histone di- and trimethylation can regulate gene transcription. To study the role of KDM4C in OCT-4 gene expression, we performed chromatin immunoprecipitation (ChIP) assays to examine whether KDM4C can directly bind to the promoter region of OCT-4 using the SK-OV-3 sphere. Indeed, our results showed that KDM4C bound to the promoter regions of OCT-4 (Figure 4(a)). In addition, our data revealed that depletion of KDM4C led to a dramatic increase in levels of di- and trimethylated H3K9 histones on the OCT-4 promoter (Figures 4(b) and 4(c)), consistent with the decreased OCT-4 expression in these spheres (Figure 4(d)).

## 3. Discussion

KDM4C, an oncogene that is frequently amplified in esophageal squamous cell carcinomas, is able to demethylate tri- and dimethylated lysine 9 on histone H3 and activates subsequent oncogenic pathways [14, 18]. Furthermore, the important role of KDM4C in CSC maintenance has made this epigenetic factor a promising target for cancer intervention [20–22].

In this study, we used the sphere culture assay to enrich the CSC population in ovarian cancer cells. We screened 21 histone demethylases and identified that KDM4C is upregulated in both SK-OV-3 and HO-8910 cells, suggesting a role of KDM4C in regulating “stemness” of ovarian cancer cells. We found that silencing of KDM4C led to repressed cell migration and invasion. Consistently, a previous study has shown that KDM4C can increase cell migration and invasion via CUL4A in lung cancer [16]. In addition, KDM4C can interact with chromosomes during mitosis to regulate the breast cell proliferation, migration, and invasion [17], suggesting a common oncogenic role of KDM4C in different cancer types.

Our results demonstrated that KDM4C is required for CSC properties. Depletion of KDM4C reduced the sphere-forming, colony-forming ability and the proportion of ALDH^high^ population in ovarian cancer cells. It has been reported that stem cell-like chromatin features in human glioblastoma CSCs are linked to a loss of the H3K9me3 mark [22]. Genetic knockout mouse model results also demonstrated that KDM4 activity is required for hematopoietic stem cell maintenance via accumulation of H3K9me3 on transcription start sites of stem cell-related genes [23]. In addition, our data revealed that KDM4C directly binds to the promoter region of pluripotency factor OCT-4 and regulates its expression. In conclusion, our data provide a novel epigenetic mechanism of CSC regulation in ovarian cancer.

## 4. Materials and Methods

### 4.1. Cell Culture

The SK-OV-3 and HO-8910 human ovarian cancer cell line was purchased from China National Infrastructure of Cell Line Resource. For the monolayer culture, cells were maintained in Dulbecco's modified Eagle's medium (DMEM, Invitrogen, China), containing 10% fetal bovine serum (FBS, Gibco, China) and 1% penicillin/streptomycin (Invitrogen, China) at 37°C under an atmosphere of 5% carbon dioxide and 95% air. For the sphere-forming assay, cells were seeded into 6-well ultralow attachment plates (Corning, China) and cultured under 1 : 1 DMEM/F12 medium-containing N2 supplement (100x, Invitrogen, China), B27 supplement (50x, Invitrogen, China), basic fibroblast growth factor (bFGF; 10 ng/ml, PeproTech, China), and human recombinant epidermal growth factor (EGF; 10 ng/ml, PeproTech, China).

### 4.2. shRNA Transfection

Lentiviral particles containing shRNA against human KDM4C and scrambled lentiviral particles were purchased from GenScript (China). In summary, SK-OV-3 and HO-8910 were cultured up to 70% confluence and were then treated with polybrene (Solarbio, China) and lentiviral particles containing shRNA against KDM4C or scrambled particles. Transfected cells were then selected using puromycin (MCE, China), and knockdown efficiency of KDM4C was assessed by qPCR and Western Blot.

### 4.3. Detection of Gene Expression by qRT-PCR and Western Blot

Quantitative reverse-transcription polymerase chain reaction (qRT-PCR) was used to determine the mRNA expression levels of genes. Briefly, total RNA was extracted from cells by using the TRIzol reagent (Invitrogen, China) according to the manufacturer's instructions. RNA was reverse transcribed to cDNA by using a Reverse Transcription Kit (Takara, China). Real-time PCR analyses were performed with Power SYBR Green (Takara, China), and the primers were synthesized at Invitrogen, China. The primers for each gene were based on a previous report [24]. Results were normalized to the expression of the human *β*-actin gene.

For the Western Blot analysis, the total protein was lysed in a radioimmunoprecipitation assay buffer (RIPA buffer, Solarbio, China). Samples were prepared under reducing conditions by using SDS-PAGE gels before being blotted and detected using an anti-KDM4C antibody (Abcam, China), OCT-4 (Abcam, China), and *α*-tubulin (Abcam, China).

### 4.4. Analysis of ALDH High Cell Subsets

An ALDEFLUOR assay kit (Stem Cell) was used to measure the aldehyde dehydrogenase (ALDH) enzymatic activity following the manufacturer's instructions. In summary, 1,000,000 cells were stained in buffer containing the ALDH substrate with or without DEAB and incubated at 37°C for 30 min. Cells were rinsed in PBS, and the fluorescence intensity was analyzed using a BD FACSCalibur flow cytometer.

### 4.5. Wound-Healing Assay, Transwell Assay, and MTT Assay

Cell migration capability was evaluated by wound-healing assay. 3 × 10^5^ cells were plated in 6-well plates for 24 h. Monolayer cells were scraped using a sterile 200 *μ*l tip, washed with cold sterile PBS to remove cell debris, and then replenished with fresh culture medium. Representative images were obtained at 0 h and 24 h at 20x magnification using a light microscope. For the transwell assay, 2 × 10^5^ cells were resuspended in serum-free DMEM medium and placed in the transwell chamber (Corning, China), while 600 *μ*l of 1% serum DMEM medium was added to the lower chamber. After incubation for 24 h, cells on the upper surface of the transwell chamber were removed with a cotton swab. The chamber was washed with PBS, fixed in precooled methanol for 5 min, and stained with 0.1% crystal violet solution for 10 min for image acquisition. MTT assays were performed using the manufacturer's guidance (Thermo Fisher, China).

### 4.6. ChIP Assays

For each ChIP reaction, 2 × 10^6^ spheroid cells were fixed in formaldehyde for 15 min at 37°C. Crosslinked chromatin was sonicated to obtain 200–500 bp fragments and immunoprecipitated using anti-KDM4C, anti-H3K27me2, or anti-H3K27me3 antibody (Abcam, China). Normal human IgG was used as a negative control.

### 4.7. Statistical Analysis

Statistical analysis in all the experiments is based on at least three biological replicates, and the error bars are drawn with the standard deviation (SD). The *P* value is calculated by using Student's *t*-test.

## Data Availability

All data are available upon request.

## Supplementary Materials

Supplementary Materials Figure S1: inhibition of KDM4C does not affect proliferation of SK-OV-3 and HO-8910 cells.
