# Supplementary figures and images for: Histone Demethylase KDM4C Is Required for Ovarian Cancer Stem Cell Maintenance
Source: Stem Cells Int. 2020 Aug 29;2020:8860185. doi: 10.1155/2020/8860185 (PMC7475738; doi:10.1155/2020/8860185)

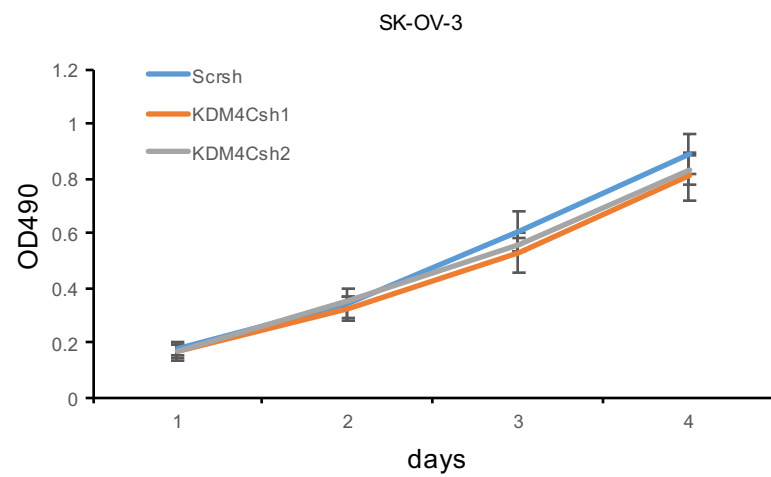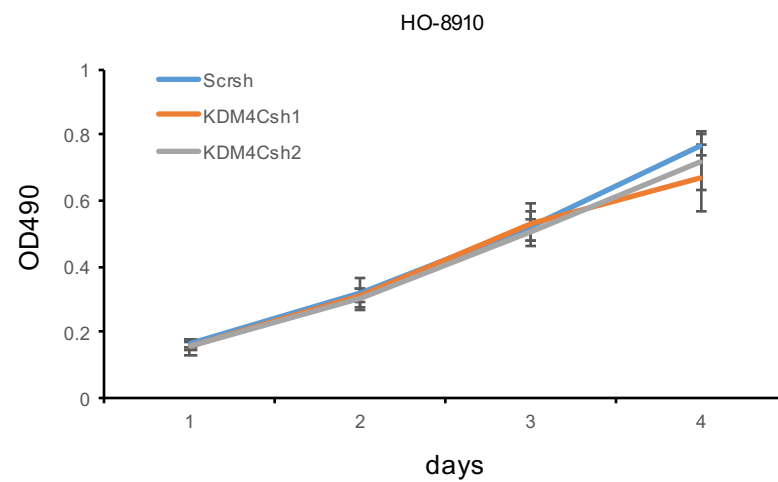

Supplement: Supplementary Materials — Figure S1: inhibition of KDM4C does not affect proliferation of SK-OV-3 and HO-8910 cells. [file 8860185.f1.pdf]
